# Does as Little as Two Hours a Day of Television Viewing Increase the Risk of Young-Onset Colorectal Cancer?

**DOI:** 10.1093/jncics/pky074

**Published:** 2019-01-25

**Authors:** J Vardy, S Kay, J Turner, H P van der Ploeg

**Affiliations:** 1 Sydney Medical School, University of Sydney, Australia; 2 Concord Cancer Centre, Concord Repatriation General Hospital, NSW, Australia; 3 Centre for Medical Psychology and Evidence-Based Decision-Making, University of Sydney, Australia; 4 Department of Public and Occupational Health, Amsterdam Public Health Research Institute, Amsterdam UMC, Vrije Universiteit Amsterdam, The Netherlands; 5 Sydney School of Public Health, University of Sydney, Australia

Colorectal cancer (CRC) is the second-most commonly diagnosed cancer and the second-most common cause of cancer-related death in the United States (1). Overall, incidence is stable, but in the last 20 years there has been an increase in young adults being diagnosed (2), the cause of which is unknown. This is particularly concerning because young-onset CRC leads to increased disease burden due to life years lost and long-term morbidity, including psychosocial and quality of life impact, as well as costs to the economy in lost productivity. It frequently presents at an advanced stage of disease with aggressive histopathology. This may be due to delays in diagnosis or perhaps a different pathophysiology in younger patients (3–5).

The study by Nguyen et al. provides important data suggesting the risk of young-onset CRC, defined as onset before 50 years of age, is greater in those with higher rates of TV viewing, after adjusting for physical activity and obesity (6). The 2018 US Physical Activity Guidelines recommend achieving 150–300 minutes of moderate to vigorous physical activity per week. Among the many health benefits, the US guidelines concluded that regular moderate-to-vigorous physical activity reduces the risk of cancers of the bladder, breast, colon, endometrium, esophagus, kidney, lung, and stomach. The report continues that the scientific evidence demonstrates that more time spent in sedentary behavior is related to greater all-cause mortality, cardiovascular mortality and incidence, and incidence of type 2 diabetes and of colon, endometrial, and lung cancer (7).

Although not specific to early-onset CRC, independent effects of sedentary behavior have been disputed by some, where associations between sitting time and cancer-related biomarkers in women (8) and between TV time and cancer mortality differ by level of physical activity, with adverse effects of sedentary behavior completely eliminated in the highest quartile of moderate-to-vigorous physical activity in a recent federated meta-analysis (9). The recently published conceptual model of movement-based terminology (10) shows how on the 24-hour clock, a change in one activity of a certain intensity level automatically results in a change of another activity of another intensity (10). Moderate-to-vigorous physical activity and sedentary behavior are part of the continuous energy expenditure spectrum that constitutes 24 hours and are separated on that spectrum by light-intensity physical activity. The 2018 US guidelines concluded that even replacing sedentary behavior with light-intensity physical activity has health benefits, but emphasized the importance of engaging in moderate to vigorous physical activity because there is a clear “dose-response” relationship between increasing the intensity of physical activity when counteracting sedentary behavior (7). Given that over 70% of American adults do not meet the 150–300 minutes of moderate-to-vigorous intensity physical activity per week guideline (11), a focus on increasing light-intensity next to moderate-to-vigorous intensity physical activity in order to replace sedentary behavior seems important to have an impact on public health (12).

It must be noted that pathways downregulated by prolonged sitting may not be the same as those upregulated by physical activity, and changes in physiological responses along the activity continuum may not be linear (13). The dysmetabolic effects on glucose and insulin of prolonged, unbroken sedentary time can be counteracted by small interruptions (14,15). However, abnormal lipid metabolism via lipoprotein lipase activity pathways associated with sedentary behavior are qualitatively different from responses to physical activity, and this may be implicated in unique, deleterious effects (16).

Molecularly distinct tumors in young CRC patients are related to epigenetics (5). Data linking exercise and altered DNA methylation suggest that epigenetic mechanisms are implicated in the protective effects of physical activity (17). Higher expression of normal gene copies could hypothetically mitigate the effects of gene mutations, but long-duration sedentary time is associated with significantly lower normal gene expression compared with higher physical activity levels. (18). Breaking up sedentary time with short bursts of activity also alters expression of skeletal muscle genes involved in cellular growth and proliferation as well as lipid and glucose metabolism (19). Just as interactions have been identified between tumor molecular markers and response to physical activity (20), early-onset CRC may represent a cluster of tumor subtypes with higher vulnerability to the negative metabolic consequences of prolonged sitting.

The study by Nguyen and colleagues found TV viewing in particular to be detrimental. TV viewing represents a specific domain of sedentary time characterized by long, uninterrupted duration with minimal energy demand. Of concern is that only 14+ h/wk (ie, 2 hours/d) was a risk factor. However, the rather low upper category of 14+ hr/wk can be considered as a limitation of the current study because variation in TV viewing in this upper category is likely to be substantial and an exponential dose response relationship beyond 14 h/wk of TV viewing has been previously shown (21). Although the study of Nguyen et al. is based on an extremely large and valuable longitudinal dataset, the relatively small number of incident cases (n = 118) of young-onset CRC provides limited statistical power for this more detailed analysis in the 14+ category as well as for other, more detailed analyses.

Of importance to note, TV viewing is no longer a common behavior for young adults with the introduction in recent years of alternative screen time devices, including smartphones and tablets. These new screen time modes also allow a higher variation in posture and movement as screen time has become substantially more mobile. Nguyen et al. identified the exclusion of these modes of sedentary behavior as a study limitation, but it highlights the need to assess these modes of screen time when measuring sedentary behavior as an outcome in future studies.

The ongoing CHALLENGE (CO21) randomized controlled trial will not only provide higher level evidence on the role physical activity plays in disease-free survival in colon cancer survivors (22), but may provide important information from its Australian substudy to determine whether a cancer recurrence is more likely in survivors with greater objectively assessed sedentary behavior, after adjusting for physical activity. However, like Nguyen’s study, the number of cases will provide limited statistical power to evaluate this in younger age onset.

Although the mechanisms by which sedentary behavior may influence CRC need to be studied more, if decreasing sedentary behavior can decrease the risk of CRC, particularly distal cancers, it provides a potential target for public health programs and interventions for all age groups. Decreasing sedentary behavior may be particularly helpful for decreasing young-onset CRC because this age group is not routinely screened for CRC. Replacing sedentary time with moderate-to-vigorous intensity physical activity is likely to be most beneficial. However, given the feasibility of replacing large volumes of sedentary time, a focus on increasing light-intensity physical activity in parallel also seems warranted.

## Notes

Affiliations of authors: Sydney Medical School, University of Sydney, Australia (JV); Concord Cancer Centre, Concord Repatriation General Hospital, NSW, Australia (JV, JT); Centre for Medical Psychology and Evidence-Based Decision-Making, University of Sydney, Australia (JV, SK, JT); Department of Public and Occupational Health, Amsterdam Public Health Research Institute, Amsterdam UMC, Vrije Universiteit Amsterdam, The Netherlands (HPvdP); Sydney School of Public Health, University of Sydney, Australia (HPvdP).
